# Editorial: The role of non-coding RNAs in gynecological cancers: new perspectives in cancer therapy and prognosis

**DOI:** 10.3389/fonc.2024.1533792

**Published:** 2024-12-10

**Authors:** Stefano Restaino, Anupa Geethadevi, Giulia Pellecchia, Anjali Geethadevi, Kaushik Das

**Affiliations:** ^1^ Clinic of Obstetrics and Gynecology, “Santa Maria della Misericordia” University Hospital Azienda Sanitaria Universitaria Friuli Centrale, Udine, Italy; ^2^ PhD School in Biomedical Sciences, Gender Medicine, Child and Women Health, University of Sassari, Sassari, Italy; ^3^ Department of Pediatric Oncology, Johns Hopkins University, Baltimore, MD, United States; ^4^ Department of Medicine - University of Udine, Udine, Italy; ^5^ Department of Obstetrics and Gynecology, Medical College of Wisconsin, Milwaukee, WI, United States; ^6^ Department of Biotechnology, Biotechnology Research and Innovation Council-National Institute of Biomedical Genomics, Kalyani, West Bengal, India

**Keywords:** gynecologic neoplasms, non-coding RNAs, tumor microenvironment, personalized medicine, cancer therapy

Gynecological cancers are the most prevalent cancers among women and exhibit a high mortality rate owing to difficulty in early diagnosis and thereby definitive treatment, with treatment resistance also adding to the challenge. Within this umbrella of tumors, endometrial cancer (EC) is among the most common while cervical (CC) and ovarian cancer (OC) are the most fatal.

Currently, research in the area is rapidly progressing not only to ascertain targetable molecules to develop viable therapeutic options for these heterogeneous tumors, but also to identify biomarkers for early diagnosis. This Research Topic features four articles that comprehensively discuss these aspects. Some authors delineated the role of ncRNAs, lncRNAs, exosomes and extracellular vescicles (EVs) in the progression of gynecological cancers, which might help both in timely diagnosis and clinical targeting (Chatterjee et al., Niebora et al., Yuan et al.), while another group elaborated the mechanisms underlying radio-resistance (Liang et al.). Understanding the importance of molecular and intercellular interactions in determining the behavior of the individual neoplasms has opened new avenues in precision medicine.

The comprehensive review by Chatterjee et al. emphasized the utility of extracellular vesicles for the early diagnosis of gynecological cancers in an attempt for their timely mitigation. EVs are nano-sized membrane-bound particles containing biomolecules, that are released into the extracellular environment by nearly all types of cells including tumor cells. The ability to isolate and quantify these EVs having tumor specific signatures could help in early detection of tumors. EV components can be associated with CC (Wnt7b mRNA and MCM3AP-AS1), EC (lncRNA TC0101441), OC (MMP-1), and uterine cancer (miR-369-3p and miR-654-3p) progression by promoting invasion, migration and angiogenesis. Reportedly, certain EVs render chemo- and radio-resistance in some cancers. Circular nc-RNAs, (hsa_circ_0109046, hsa_circ_0002577) appear as predictive biomarkers of EC. In multiple cancer types, EVs isolated from cervicovaginal fluid, plasma and/or serum of affected patients containing various miRNAs and secretory proteins can be promising biomarkers that could help in timely diagnosis. Bioengineered EVs can be utilized as delivery tools to transport specific biomolecules that effectively target tumor cells without inciting host immune response. Bone marrow mesenchymal stem cell (BMMSCs) derived EVs containing miRNAs (miR-144-3p or miRNA-331-3p) and even chemotherapeutic drugs (cisplatin and paclitaxel) has been shown to mitigate CC and EC respectively, *in vitro* as well as in tumor xenograft models. The authors emphasize the importance of investigating these EVs in the light of tumor heterogeneity, which will lay the foundations for personalized therapies in the future.

Niebora et al. contributed to this Research Topic with an interesting overview about the role of ncRNAs in EC. The authors highlight the crucial role that exosomes play in regulating the tumor microenvironment and thereby tumor progression. Exosomes are EVs containing DNA or RNA and mediate intercellular communication between EC cells, tumor-associated fibroblasts and tumor-associated macrophages. MiR-93, miR-21, miR-133a increase tumor proliferation, metastasis and aggressiveness conferring poor prognosis for the patient. Induced expression of lncRNA GAS5 and miRNAs, miR-192-5p and miR-503-3p might be helpful in targeting ECs through induction of apoptosis and tumor suppression. High expression of LncRNA BMPR1B-AS1 in EC compared to normal tissues could drive aggressive behaviors in EC cells by activating the cell cycle and enhancing mesenchymal characteristics. Overexpression of lncRNA HEIH could contribute to the resistance of EC cells to paclitaxel by enhancing MAPK signaling. The authors present valid arguments that might pose additional challenges to the use of ECs on the clinical front. These include genetic variability, change in marker levels at different stages of the disease as well as in various post-treatment stages. This warrants studies that are more comprehensive, taking into consideration a large and heterogeneous patient population as well as stratification based analysis to include disease stages, treatment type and other pathophysiological aspects.

Yuan et al. showed the association of the expression levels of a few critical genes with the clinico-pathological features of cervical squamous cell carcinoma (CSCC). They found that the transcript expression of lnc-CCDC170-4:1 was low in CSCC and is associated with lymph node metastasis and tumor size. LncRNA SRA, known to induce EMT, was high in CSCC and strongly associated with FIGO staging which complements previous studies that link this lncRNA with CC progression and aggressiveness. Interestingly, CSCC had low ESR expression, which was associated with FIGO Staging and Tumor size accompanied by a high expression of aromatase.

The comprehensive review from Liang et al. emphasized the important aspect of gynecological cancer management, which is, treatment resistance. They discussed factors like DNA damage repair, changes in tumor microenvironment, hypoxia, cancer stem cell (CSC) generation and epigenetic factors to be key in rendering radio-resistance in CCs. Altering some components of DNA repair pathways like knocking out MTDH, downregulation of TRIP4 or overexpression of HNF1A may help sensitize CC to radiation therapy. The authors also described the processes by which the interaction between tumor fibroblasts, tumor macrophages and tumor cells occur via autocrine and paracrine mechanisms activated by radiation exposure, which initiate a vicious cycle that leads to radio-resistance. CSCs are the self-renewing population of tumor cells and are widely recognized for their ability to render treatment resistance in various cancers. Inhibiting miRNA125 significantly improves chemo- and radio-sensitivity in CC by inhibiting the CSC phenotype. Further, miRNAs and their regulation can trigger radiation-associated signaling pathways, change the progression of the cell cycle, influence DNA repair mechanisms, and thereby affect the emergence, growth, and response of tumors to radiotherapy. The authors also meticulously discussed various DNA and histone modifications that significantly contribute to radio-resistance in CC, implying that epigenetic drugs could prolong remission in combination with standard therapies. The authors further elaborated on the potential of non-coding RNAs in the pathogenesis of CC, which have great potential to be used for therapy, however more methodical studies are needed for clinical translatability.

In summary, all the articles featured in this Research Topic bear the potential to expand our knowledgebase about the current status of research in gynecological cancers. These studies would help pave the way for important research breakthroughs, leading to advancements in early diagnosis and effective personalized therapies for gynecological cancers.

